# A human rights-based approach to the reimbursement of expensive medicines

**DOI:** 10.2471/BLT.15.166371

**Published:** 2016-11-10

**Authors:** Katrina Perehudoff, Brigit Toebes, Hans Hogerzeil

**Affiliations:** a University Medical Centre, University of Groningen, Ant Deusinglaan 1, Building 3217 ('de Brug'), 9713 AV Groningen, Netherlands.; b Global Health Law Groningen Research Centre, Department of International Law, University of Groningen, Groningen, Netherlands.

In recent years, pharmaceutical companies have introduced new expensive medicines, some of which target only small patient populations. For example, trastuzumab (Herceptin®), imatinib (Glivec®) and sofosbuvir (Sovaldi®) are high-priced medicines that have been shown to be effective and safe for treating cancer or hepatitis C, diseases for which no effective treatment existed previously. In 2015, the World Health Organization included several such expensive medicines in their model list of essential medicines,^1^ despite current prices of 60 000–100 000 United States dollars per treatment. The initial public enthusiasm for these medicines’ therapeutic value is now tempered by practical concerns about how patients and health systems can afford them. Even in high-income countries the affordability is a concern.

Ethical and economic dilemmas arise when decision-makers must ration an expensive, life-saving or life-extending medicine. We contend that not funding an effective essential medicine contradicts Article 12 in the International Covenant on Economic, Social and Cultural Rights, which declares 

“the States Parties to the present Covenant recognize the right of everyone to the enjoyment of the highest attainable standard of physical and mental health.” 

By September 2016, over 160 countries have ratified or acceded to the Covenant.^2^ In many countries, such as Argentina, Brazil, Latvia, New Zealand and Uruguay, patients have used Article 12 to litigate through domestic courts for the reimbursement of expensive medicines.^3^ In the Netherlands, advocates of patients with Pompe’s disease – a rare muscle disease – based part of their arguments on the fact that it was the patients' right to receive continued reimbursement of an expensive treatment with marginal health benefits. Decision-makers decided to continue reimbursement, despite the fact that many other patients in the country were not reimbursed for more cost–effective treatments.

How can governments with finite public health budgets ensure fair access to expensive, new and essential medicines as part of the right to health? Is the right to health equally important for all patients?

Governments’ efforts to prioritize access to essential medicines could be supported by a human rights approach and specifically by the principle of the progressive realization of the right to health.^2^ The United Nations Committee on Economic, Social and Cultural Rights describes progressive realization as States Parties’ obligation to use the maximum of its available resources to move as expeditiously and effectively as possible towards achieving the highest attainable standard of health for everyone.^2^^,^^4^ The approach does not necessarily create an immediate right for everyone to receive treatment at any cost. Instead, progressive realization means that access to health care is gradually fulfilled for all patients. All Parties have an immediate obligation to take deliberate, concrete and targeted steps towards progressive realization.^5^ However, providing immediate universal health care with all possible treatments for all people is not a legal obligation.^5^

For prioritization, a transparent and independent reimbursement ranking process of medical treatments, largely based on estimates of comparative cost–effectiveness, presents a concrete and practical tool for decision-makers. Progressive realization justifies such ranking of treatments by ensuring that governments use available resources as effectively as possible and thereby progressively realizing the right to health for the largest number of people at the lowest possible cost and without discrimination against patients. This duty of efficiency is also in line with the authoritative interpretation by the Committee on Economic, Social and Cultural Rights of governments’ legal obligations under the human rights treaties in General Comment 3, as well as a well-founded set of principles adopted by a group of scholars and policy-makers in the Limburg Principles.^4^^,^^6^

Progressive realization also presents a human rights justification for not reimbursing certain treatments, thereby protecting fair reimbursement decisions from being derailed by patients claiming very expensive treatments based on their individual right to health, in the absence of other criteria, such as comparative cost–effectiveness. An argument to claim reimbursement of very expensive treatments is that patients with rare diseases are disadvantaged because fewer patients need to cover the development costs of the medicine. In 2005, McCabe et al. reviewed and dismissed this argument. They based their arguments on the fact that the public sector often already invests substantially in orphan drug development.^7^ Furthermore, the authors questioned whether the public sector should also subsidize the private sector to develop orphan medicines that will cost society more than the health benefits to be gained. From a human rights perspective, the key message to those patients is that their right to health is recognized, but that it can only be fulfilled in the future. The fact that a disease is rare is, in itself, not a justification for unlimited reimbursement.

To accelerate progressive realization and increase the number of patients that are eligible for reimbursement, governments could increase their health budgets, an action supported by the Parties’ obligation to use the maximum of their available resources.^2^ A much more effective way to realize the right to health would be through targeted government action using all available tools to reduce prices, such as the flexibilities to the *Agreement on trade-related aspects of intellectual property rights*.^8^ Some new medicines, such as antiretroviral therapy, were initially marketed at very high prices but became much cheaper after targeted price-reduction actions from governments.^9^

Pharmaceutical companies and their shareholders also have a duty under human rights law to ensure that their new products are made available and affordable to all who need them.^10^ They should, therefore, refrain from actions that limit accessibility, such as pursuing stronger intellectual property protection, and should, within a viable business model, ensure that new medicines are accessible to all those in need.^10^ In addition, the publication *Guiding Principles on Business and Human Rights* advocates that companies respect the right to health and access to medicines.^11^ Companies’ violations of these human rights principles give national governments a justification to impose corrective measures, such as compulsory licencing for cheaper domestic production. For example, the anti-cancer drug sorafenib tosylate (Nexavar®) from Bayer is now produced in India at a more affordable price because of a compulsory licence issued by the government.^12^

Finally, a transparent and independent reimbursement ranking process is important to show that all patients are treated equally and without discrimination. The process should adopt explicit criteria, such as comparative cost–effectiveness, severity of the disease or end-of-life care, which should be based on standards set independently of the pharmaceutical industry. The weight of these criteria in the ranking process should be established in consultation with beneficiaries and the criteria should be adjudicated consistently. Such transparent decision-making can inspire meaningful and balanced public consultation and can better inform the public debate on trade-offs during the ranking process.^13^ Such transparency also promotes greater accountability of governments for their reimbursement decisions. The use of the Accountability for Reasonableness Framework can further guide the fair rationing of health care.^14^
